# Dactylitis Syphilitica

**Published:** 1871

**Authors:** 


### *Dactylitis Syphilitica.*

We have just received an exceedingly interesting monograph of thirty pages on Dactylitis Syphilitica, with observations on Syphilitic Lesions of the Joints, by R. W. Taylor, M.D., Surgeon to the New York Dispensary, Department of Venereal and Skin Diseases.

After alluding to the more superficial syphilitic lesions of the fingers and toes as palmar and plantar psoriasis and non-ulcerating tuburcular syplilides, he says:

It is not intended, however, to enter fully into the consideration of these more superficial lesions, but to present the clinical history of a peculiar and rare manifestation of syphilis in the deeper structures of the fingers and toes. This affection consists in the deposit of the peculiar gummy material of tertiary syphilis in one or all of the deep tissues, and is characterized by peculiar deformities. Its literature is very scanty in details, and of quite recent origin. It has been called, by some observers, syphilitic panaris, and by others, syphilitic dactylitis. The application of the word panaris, which is merely a corruption of the word paronychia, to the affection is neither appropriate nor expressive, as it applies to a simple acute inflammation of that portion only of the fingers upon which the nails are situated; whereas the lesion under consideration is a chronic specific inflammation generally involving more than one, and frequently all the phalanges without any affection of the nails. The word dactylitis, from the Greek, a digit or finger, fully expresses the condition: therefore its use will be retained here, as it applies with equal correctness to the fingers and the toes.

We have read with great interest this paper, and feel that Dr. Taylor deserves the thanks of the entire profession for this valuable contribution.

